# Reciprocal transactivation of Merkel cell polyomavirus and high-risk human papillomavirus promoter activities and increased expression of their oncoproteins

**DOI:** 10.1186/s12985-021-01613-0

**Published:** 2021-07-03

**Authors:** Kashif Rasheed, Baldur Sveinbjørnsson, Ugo Moens

**Affiliations:** 1 Molecular Inflammation Research Group, Department of Medical Biology, Faculty of Health Sciences, University of Tromsø, The Arctic University of Norway, 9037 Tromsø, Norway; 2 Childhood Cancer Research Unit, Department of Women’s and Children’s Health, Karolinska Institutet, 1176 Stockholm, Sweden; 3 Present Address: Institute for Clinical and Molecular Medicine, Norwegian University of Science and Technology, NTNU Trondheim, Trondheim, Norway

**Keywords:** Co-infection, Promoter, Tumor, Luciferase assay, Cervical cancer

## Abstract

**Background:**

Approximately 15% of human cancers are attributed to viruses. Numerous studies have shown that high-risk human polyomaviruses (HR-HPV) and Merkel cell polyomavirus (MCPyV) are two human tumor viruses associated with anogenetal and oropharyngeal cancers, and with Merkel cell carcinoma, respectively. MCPyV has been found in HR-HPV positive anogenetal and oropharyngeal tumors, suggesting that MCPyV can act as a co-factor in HR-HPV induced oncogenesis. This prompted us to investigate whether the oncoproteins large T-antigen (LT) and small antigen (sT) of MCPyV could affect the transcriptional activity HPV16 and HPV18 and vice versa whether HPV16 and HPV18 E6 and E7 oncoproteins affected the expression of MCPyV LT and sT. Reciprocal stimulation of these viral oncoproteinscould enhance the oncogenic processes triggered by these tumor viruses.

**Methods:**

Transient co-transfection studies using a luciferase reporter plasmid with the long control region of HPV16 or HPV18, or the early or late promoter of MCPyV and expression plasmids for LT and sT, or E6 and E7, respectively were performed in the HPV-negative cervical cancer cell line C33A, in the keratinocyte cell line HaCaT, and in the oral squamous cell carcinoma cell line HSC-3. Transfections were also performed with deletion mutants of all these promoters and with mutants of all four oncoproteins. Finally, the effect of E6 and E7 on LT and sT expression in the MCPyV-positive Merkel cell carcinoma cell line WaGa and the effect of LT and sT on the expression of E6 and E7 was monitored by Western blotting.

**Results:**

LT and sT stimulated the transcriptional activity of the HPV16 and HPV18 LCR and v.v. E6 and E7 potentiated the MCPyV early and late promoter in all cell lines. Induction by E6 and E7 was p53- and pRb-independent, and transactivation by LT did not require DNA binding, nuclear localization and HSC70/pRb interaction, whereas sT stimulated the HPV16/18 LCR activity in a PP2A- and DnaJ-independent manner.

**Conclusions:**

These results indicate that the co-infection of MCPyV may act as a co-factor in the initiation and/or progression of HPV-induced cancers.

**Supplementary Information:**

The online version contains supplementary material available at 10.1186/s12985-021-01613-0.

## Background

*Papillomaviridae* and *Polyomaviridae* are two families of non-enveloped, double-stranded DNA viruses that can cause cancer in humans [1]. High-risk human papillomaviruses (HR-HPV) are predominantly associated with cervical cancer, but also with penile, anal, vulvar, vaginal and oropharyngeal cancers [2–10]. HR-HPV16 and HR-HPV18 are responsible for almost 70% of cervical cancers worldwide, and integration of the viral genome is considered one of the most important risk factors for cervical cancer development [5].The oncogenic potentials of HR-HPVs mainly depend on their oncoproteins E6 and E7. These proteins can bind p53 and pRb family members, respectively, but can also interfere with other hallmarks of viral oncogenesis [11–13]. Merkel cell polyomavirus is an etiological factor for Merkel cell carcinoma (MCC), which is an aggressive skin cancer [14]. Approximately 80% of all MCC are positive for MCPyV DNA [15], and similarly to HR-HPV in cervical cancer, the MCPyV genome is integrated into all virus-positive MCC examined so far [16, 17]. The viral oncoproteins of MCPyV are denoted as large tumor antigen (LT) and small tumor antigen (sT) [18, 19]. Equivalently, as seen in HPV, LT can interfere with the activity of p53 and pRb proteins [19, 20].This similar effect also suggests that E6, E7 and LT may act together to augment the transformative properties of HR-HPV in cells were co-infection occurs.

Co-infection of human polyomaviruses in HR-HPV positive tumors has been reported, underscoring the possibility that human polyomaviruses can act as a co-factor in HR-HPV-induced oncogenesis. Co-presence of BK polyomavirus (BKPyV) and HR-HPV has been reported in oropharyngeal malignancies, cervical swabs, and genital tumors [2, 21–25]. BKPyV DNA or JC polyomavirus DNA was detected in HR-HPV positive precancerous cervical lesions and in oropharyngeal squamous cell carcinomas [22, 26, 27]. However, whether these polyomavirus have an impact on cancer development remains unknown.

In cervical tumor samples, MCPyV DNA could be amplified in 19% of HR-HPV positive cervical squamous cell carcinomas (n = 48), and in 25% of HR-HPV positive cervical adenocarcinomas (n = 16) [28]. In another study, 14 out of 45 (34%) HR-HPV positive cervical tumors were also positive for MCPyV DNA [29]. Kolia-Diafouka and colleges detected HR-HPV in 124 out of 140 (88.6%) cervical samples from HIV-positive African women and in 24 out of 50 (48%) samples from HIV-negative French women. Interestingly, 81 (55%) of these 148 HR-HPV positive samples were also positive for MCPyV, but there was no significant difference with HR-HPV negative samples, of which 24/42 (57%) were MCPyV positive. There was no association between detection of human polyomaviruses in cervical specimens and HR-HPV coinfection or precancerous cervical lesions [30]. MCPyV DNA has also been observed in HR-HPV positive oropharyngeal cancers and in HR-HPV positive tonsillar SCC, but the prevalence of MCPyV DNA in benign tonsillar tissue did not differ significantly compared with malignant tissue [24, 31, 32]. These observations argue against a possible role of MCPyV as a cofactor in HPV-induced carcinogenesis.

Although a co-factor role for MCPyV in HR-HPV-induced cancers remains to be proven, the co-detection of MCPyV DNA in HR-HPV positive tumor specimens and the known oncogenic potential of MCPyV suggest that MCPyV may promote HPV-induced oncogenesis. This prompted us to investigate whether the MCPyV oncoproteins LT and sT could enhance the expression of the major HR-HPV oncoproteins E6 and E7, thereby accelerating the initiation and progression of HR-HPV-induced neoplasia. A possible effect of E6 and E7 on the transcriptional activity of MCPyV was also explored.

## Methods

### Cell lines

The human cell line C33A (human papilloma virus-negative cervical cancer cells) was purchased from the American Type Culture Collection (ATCC, Manassas, VA, USA; cat. no. HTB-31) and HaCaT human keratinocyte cell lines were obtained from Cell Lines Services, (Eppelheim, Germany; cat. no. 300493). Both cell lines were maintained in Dulbecco’s Modified Eagle’s Medium (DMEM; Sigma D5796) with 10% fetal bovine serum (Life Technologies, cat. no. 10500-064). HSC-3 cells, established from human oral squamous cell carcinoma from the tongue, were a kind gift from Dr. Gunbjørg Svineng (University of Tromsø) and were cultivated in 1:1 DMEM/F-12 medium supplemented with 50 μg/ml ascorbic acid (Sigma-Aldrich), 0.4 μg/ml hydrocortisone (Sigma-Aldrich) and 10% fetal bovine serum. Cells were kept at 37 °C in a humidified CO_2_ incubator.

### Plasmids

The HPV16 luciferase reporter plasmid (pGL4-LCR-HPV16-LCR (nt 7000-100)-luciferase) and the HPV18 luciferase reporter plasmid (pGL4.20 HPV18-LCR luciferase) were obtained from Addgene (p5193 and p5194, respectively; Watertown, MA, USA). The HPV16 E6 and E7 expression plasmids were purchased from Addgene (p1322 and p1324, respectively). The luciferase reporter plasmid with the HPV16 long control region (LCR) contains nucleotides 6138 to 7131 (GenBank accession NC_001526; [33]), whereas the luciferase reporter plasmid with the HPV18 LCR encompasses nucleotides 6943 to 105 (GenBank accession X05015; [34]). Using these plasmids, we analyzed luciferase activity driven by these LCR. The empty expression vector pcDNA3.1( +) was purchased from Thermo Fisher Scientific (Waltham, MA, USA). The MCPyV LT expression vector was obtained from Addgene (p28189), while the MCPyV sT expression plasmid was a kind gift from Dr. Andrew Macdonald [35]. The expression vectors for MKL1 and MKL2 LT have been previously described [36]. The lentiviral plasmids pLJM1-HPV16-LCR-EGFP and pLJM1-HPV16-LCR-EGFP were constructed by replacing the CMV promoter in pLJM1-EGFP (Addgene, #19,319) with the HPV16 or HPV18 LCR, respectively using *SnaB*I and *Afe*I restriction sites.

### Generation of mutant E6, E7, LT, and sT expression plasmids by site-directed mutagenesis

PCR based site-directed mutagenesis using the primers shown in Table 1 was used to generate different mutant E6, E7, LT and sT expression plasmids. QuickChangeTM site-directed mutagenesis kit from Aligent Technologies (Santa Clara, CA, USA) was used according to the manufacturer’s instruction. All mutations were confirmed by sequencing using the BigDye Terminator v3.1 Cycle Sequencing kit (ThermoFisher Scientific; cat. no. 4337455) and the ABI 35000xL (Applied Biosystems, Foster City, CA, USA).

**Table 1:** Sequences of primers used in this study

Name	Sequence (5′-3′)	Purpose	References
HPV16 E6_F47R.Fw	GGTATATGACTTTGCTCGTCGGGATTTATGC	defective for polyubiquitination	[48]
HPV16 E6_F47R.Rv	GCATAAATCCCGACGAGCAAAGTCATATACC	defective for polyubiquitination	[48]
HPV16 E6_C106R.Fw	GGTGTATTAACCGTCAAAAGCCACTG	p53 binding mutant	[47]
HPV16 E6_C106R.Rv	CAGTGGCTTTTGACGGTTAATACACC	p53 binding mutant	[47]
HPV16 E7_AGQ.Fw	CCAGAGACAACTGATGCCTACGGTTATCAGCAATTAAATGACAGC	pRb binding mutant	[49]
HPV16 E7_AGQ.Rv	GCTGTCATTTAATTGCTGATAACCGTAGGCATCAGTTGTCTCTGG	pRb binding mutant	[49]
HPV16 E7_GSE.Fw	GTAACCTTTGGTAGCGAGTGTGACTCTACG	CxxC mutant	[12]
HPV16 E7_GSE.Rv	CGTAGAGTCACACTCGCTACCAAAGGTTAC	CxxC mutant	[12]
sT_R7A.Fw	AGTCCTAAATGCGAAAGAAAGAGAGGC	PP2A binding mutant	[45]
sT_R7A.Rv	GCCTCTCTTTCTTTCGCATTTAGGACT	PP2A binding mutant	[45]
sT/LT_D44N.Fw	GCATCACCCTAATAAAGGGGGAAATCC	DnaJ binding mutant	[45]
sT/LT_D44N.Rv	GGATTTCCCCCTTTATTAGGGTGATGC	DnaJ binding mutant	[45]
sT_L142A.Fw	GCAAAAAAACTGTGCGACGTGGGGAGAG	PP2A binding mutant	[45]
sT_L142A.Rv	CTCTCCCCACGTCGCACAGTTTTTTTGC	PP2A binding mutant	[45]
sT_91AAAAA95.Fw	CCTTGGGAAGAATATGGAACTGCAGCGGCTGCTGCGCAAAGTGGATATAATGCTAG	Fbxw7 binding mutant	[45]
sT_91AAAAA95.Rv	CTAGCATTATATCCACTTTGCGCAGCAGCCGCTGCAGTTCCATATTCTTCCCAAGG	Fbxw7 binding mutant	[45]
LT_W209A.Fw	CGTATGGCACCGCGGAGGATCTCTTCTGC	hVam6p mutant	[44]
LT_W209A.Rv	GCAGAAGAGATCCTCCGCGGTGCCATACG	hVam6p mutant	[44]
LT_E216K.Fw	GGATCTCTTCTGCGATAAATCACTTTCCTCCCCTGAG	pRb binding mutant	[44]
LT_E216K.Rv	CTCAGGGGAGGAAAGTGATTTATCGCAGAAGAGATCC	pRb binding mutant	[44]
HPV16LCR Δ1_Fw	GTGTATATGTTTGTATGAGCTCGTATGTGCTTG	Truncated HPV16 LCR	This study
HPV16LCRΔ1_Rv	CAAGCACATACGAGCTCATACAAACATATACAC	Truncated HPV16 LCR	This study
HPV16LCRΔ2_Fw	CACCTACTAATTGAGCTCTGGTTATTCATTG	Truncated HPV16 LCR	This study
HPV16LCRΔ2_Rv	CAATGAATAACCAGAGCTCAATTAGTAGGTG	Truncated HPV16 LCR	This study
HPV16LCRΔ3_Fw	CCTGACCTGCAGAGCTCGCCAACCATTCC	Truncated HPV16 LCR	This study
HPV16LCRΔ3_Rv	GGAATGGTTGGCGAGCTCTGCAGGTCAGG	Truncated HPV16 LCR	This study
HPV18LCRΔ1_Fw	GTCCTGTGTTTGAGCTCGTTGTATGATTGC	Truncated HPV18 LCR	This study
HPV18LCRΔ1_Rv	GCAATCATACAACGAGCTCAAACACAGGAC	Truncated HPV18 LCR	This study
HPV18LCRΔ2_Fw	CCTCCATTTTGAGCTCCAACCGATTTCG	Truncated HPV18 LCR	This study
HPV18LCRΔ2_Rv	CGAAATCGGTTGGAGCTCAAAATGGAGG	Truncated HPV18 LCR	This study
HPV18LCRΔ3_Fw	CCTGTCCAGGTGAGCTCCAACAATTGCTTGC	Truncated HPV18 LCR	This study
HPV18LCRΔ3_Rv	GCAAGCAATTGTTGGAGCTCACCTGGACAGG	Truncated HPV18 LCR	This study
MCPyV-EΔ1_Fw	GTTTATCAGTCGAGCTCCGCCTCTCC	Truncated early MCPyV promoter	This study
MCPyV-EΔ1_Rv	GGAGAGGCGGAGCTCGACTGATAAAC	Truncated early MCPyV promoter	This study
MCPyV-EΔ2_Fw	GGCAGTATCTAAGGGGAGCTCCCAAGGGC	Truncated early MCPyV promoter	This study
MCPyV-EΔ2_Rv	GCCCTTGGGAGCTCCCCTTAGATACTGCC	Truncated early MCPyV promoter	This study
MCPyV-LΔ1_Fw	CAGAGGCCTCGGAGCTCAGGAGCCCCAAGC	Truncated late MCPyV promoter	This study
MCPyV-LΔ1_Rv	GCTTGGGGCTCCTGAGCTCCGAGGCCTCTG	Truncated late MCPyV promoter	This study
MCPyV-LΔ2_Fw	CCTGGAGAGGCGGAGCTCGACTGATAAACAAAAC	Truncated late MCPyV promoter	This study
MCPyV-LΔ2_Rv	GTTTTGTTTATCAGTCGAGCTCCGCCTCTCCAGG	Truncated late MCPyV promoter	This study

### Transfection

Cells were seeded out in 12-well cell culture plates so that they were approximately 70% confluent the next day when they were transfected. JetPrime (Polyplus-transfection, Illkirch, France) was used as the transfection reagent, with cells transfected with 400 ng luciferase plasmid according to the manufacturer’s instructions. In the experiments in which the effect of LT or sT on promoter activity was monitored, co-transfection was performed with increasing amounts of LT and/or sT expression plasmid being used. The amount of DNA in the different experiments was kept constant by adding the appropriate amount of empty vector pcDNA3.1( +). Each transfection was repeated at least once.

### Firefly luciferase assay

Cells were lysed 24 h post-transfection in 100 μl of Luciferase Assay Tropix Lysis solution (Applied Biosystems, Foster City, CA, USA), with 0.5 mM DTT (Sigma-Aldrich Norway AS) freshly added. Cells were scraped and transferred to Eppendorf tubes, followed by 3 min of centrifugation at 12,000*g*. Twenty μl of supernatant were transferred to a 96-well microtiter plate, and luciferase buffer (Promega, Madison, WI, USA) was added. Light units were measured using the CLARIONstar Plus Microplate Reader (BMG Labtech GmbH, Ortenberg, Germany). The luciferase values of each sample were corrected for protein concentration, as measured by the Protein Quantification Assay from Macherey–Nagel (Düren, Germany) according to the instructions of the manufacturer. OD570 was measured using the CLARIOstar Plus Microplate Reader. We corrected luciferase values by measuring the protein concentration in the corresponding sample rather than co-transfection with a *Renilla luciferase* reporter plasmid to avoid promoter interference between the HPV promoter directing expression of the firefly *luciferase* gene and a promoter controlling expression of the *Renilla luciferase* gene. In addition, many of our transfection studies include co-transfection with LT and/or sT expression plasmids, containing the strong competing cytomegalovirus (CMV) promoter. Moreover, LT of polyomaviruses have shown activate many promoters, including the SV40 promoter and the herpes simplex virus *thymidine kinase* promoter, which are used in *Renilla luciferase* reporter plasmids [37].

### Western blotting

Cells were plated out in 6-well plates and transfected the next day with 2 μg of DNA (either empty vector pcDNA3.1 or LT and/or sT expression plasmid) using JetPrime. Twenty-four hours after transfection, the cells were briefly washed with phosphate-buffered saline (PBS, Biochrom GmbH), and harvested in NuPage LDS sample buffer (Invitrogen, Carlsbad, CA, USA) with 100 mM of DTT. The samples were then sonicated and heated for 10 min at 70 °C. Proteins were separated on NuPAGE™ Novex™ 4–12% Bis–Tris Protein Gels (Thermo Fisher Scientific Inc.) and transferred onto a 0.45 μm PVDF Membrane (Merck Life Science AS, Oslo, Norway). The membrane was blocked in TBST (Tris-buffered saline with 0.1% Tween-20; Sigma-Aldrich Norway AS) containing 5% (w/v) skimmed milk powder. Antibodies used in this study were HPV16 E6 antibody (Santa Cruz Biotechnology Inc., Dallas TX, USA; cat. no. sc-460), HPV18 E6 antibody (Santa Cruz; cat. no. sc-365089), HPV16 E7 (Santa Cruz; cat. no. sc-65711), HPV18 E7 antibody (Santa Cruz; cat. no. sc-365035), rabbit anti-GFP antibody (Abcam; cat. no. ab290), mouse anti-GAPDH antibody (0411) (Santa Cruz; cat. no. sc-47724), mouse Anti-MCPyV large T-antigen Antibody (CM2B4) (Santa Cruz; cat. no. sc-136172) and mouse anti-FLAG® M2 monoclonal antibody (Agilent Technologies; cat. no. 200472). Incubation with the primary was done overnight at 4 °C in blocking buffer. Following three washes in TBST, the membrane was incubated with the polyclonal swine anti-rabbit secondary antibody conjugated with an alkaline phosphatase (D0306, Dako, Santa Clara, CA, USA) solution for 1 h at room temperature. After four washes, detection and visualization were performed using CDP-Star chemiluminiscent (C0712, Sigma-Aldrich) and the ImageQuant LAS 4000 imager (GE Healthcare, Oslo, Norway). GAPDH levels served as loading control. MagicMark™ XP Western Protein Standard (Thermo Fisher Scientific Inc.) was used to estimate the molecular mass of the detected proteins.

### Lentivirus production, transduction and stable cell lines establishment

For the lentivirus production, pLJM1-HPV16 LCR-EGFP, pLJM1-HPV18 LCR-EGFP or pLJM1-EGFP plasmids were co-transfected along with helper plasmids (pHCMV-G, pRSV rev and pMDLg/pRRE) in HEK293T cells using Lipofectamine 3000 transfection reagent (Thermofisher; cat. no. L300001). Virus supernatants were collected 24 h after transfection and filtered through 0.45-μm filters. For transduction, virus supernatants were added along with 1 μg/ml polybren to HaCaT cells and incubated overnight. The next day, the cells were washed twice with culture medium. For generation of stable cells, 1 μg/ml puromycin was applied for 7 days to select transduced cells.

### Statistical analysis

The *t* test was employed to determine statistical differences between the promoter activities.

## Results

### Transactivation of the HPV16 and HPV18 long control region activity by MCPyV LT and sT

**Fig. 1:**
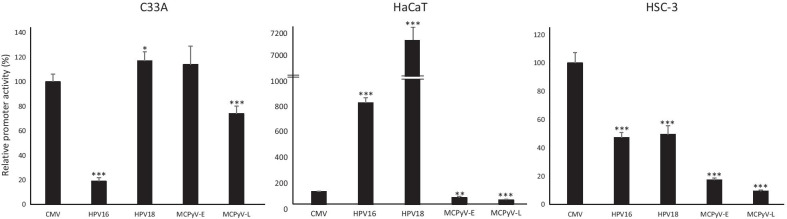
Viral promoter activities in different cell lines. The promoter activities of the CMV major immediate early promoter (CMV), the HPV16 long control region (HPV16), the HPV18 long control region (HPV18), the MCPyV early promoter (MCPyV-E), and the MCPyV late promoter (MCPyV-L) were compared in three different cell lines. Cells were transfected with 400 ng luciferase reporter plasmid and cell lysates were prepared 24 h after transfection. Luciferase activity was corrected for the protein concentration in the lysate. Each bar represent the average of three independent parallels ± standard deviation (SD). The activity of the CMV promoter was arbitrary set as 100%. Similar results were obtained in an independent experiment. *p < 0.05; **p < 0.01; ***p < 0.001

HR-HPVHPV16, HPV18 and MCPyV can be detected in cervical cancer and oropharyngeal cancers and HPV16 and HPV18 can infect keratinocytes, whereas keratinocytes have been suggested as a cell of origin of MCPyV-positive Merkel cell carcinoma [38, 39]. We therefore compared the transcriptional activities of the HPV16 LCR, the HPV18 LCR and the MCPyV early (MCPyV-E) and late (MCPyV-L) promoter in the HPV-negative cervical cancer cell line C33A, in the human keratinocyte cell line HaCaT, and in HSC-3 cells established from a human oral squamous cell carcinoma from the tongue using luciferase reporter plasmids. Because co-transfections with expression plasmids for the oncoproteins LT, sT, E6 and E7 containing the CMV major immediate early promoter were performed, promoter interference can occur [40]. Hence, we also included a luciferase reporter plasmid with this CMV promoter. The HPV16 and HPV18 LCR had comparable activity in HSC-3 cells, but the HPV18 LCR had sixfold to tenfold higher activity than HPV16 LCR in C33A and HaCaT cells, respectively (Fig. 1). The MCPyV early promoter was slightly stronger than the late promoter in the three cell lines. The strength of the CMV promoter was comparable with the other viral promoters in C33A cells, except for HPV16 LCR promoter activity, which was weaker. The CMV promoter activity was weaker than that of the HPV16 and HPV18 LCR, and slightly, but significantly stronger than the MCPyV early and late promoter activities in HaCaT cells. The CMV promoter was the strongest of all viral promoters in HSC-3 cells (Fig. 1). The promoter activity of HPV16 LCR was higher in HaCaT cells than in HSC-3 cells and C33A cells, respectively, whereas HPV18 LCR had the highest activity in HaCaT cells followed by C33A cells and then HSC-3 cells. The activities of MCPyV early and late promoter were highest in HaCaT cells, followed by C33A cells and then HSC-3 cells.

**Fig. 2:**
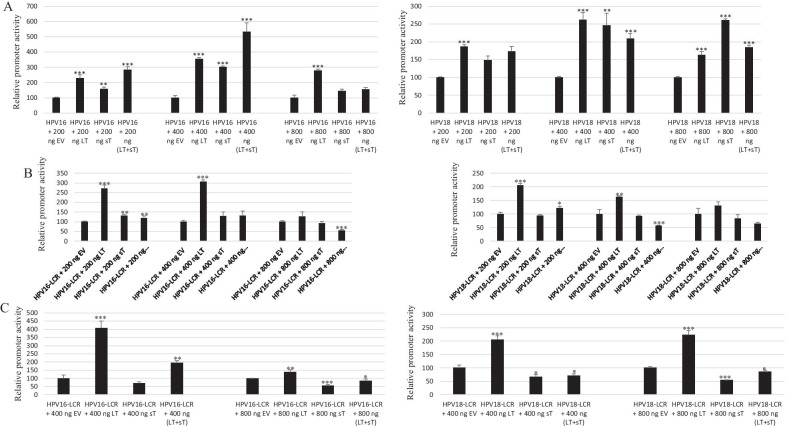
MCPyV LT and sT transactivate the transcriptional activity of HPV16 LCR and HPV18 LCR. **A** C33A cells were transfected with 400 ng luciferase reporter plasmid and increasing amounts (200 ng, 400 ng, or 800 ng) of expression plasmids for MCPyV LT or sT. **B** HSC-3 cells were transfected with increasing amounts of expression plasmids for MCPyV LT or sT. **C** HaCaT cells were transfected with increasing amounts of expression plasmids for MCPyV LT or sT. Luciferase activity was corrected for the protein concentration in the lysate. Each bar represents the average of three independent parallels ± SD. The transcriptional activity of the HPV16 LCR (respectively HPV18 LCR) in the presence of empty vector pcDNA3.1 (EV) was arbitrary set as 100%. Similar results were obtained in an independent experiment. *p < 0.05; **p < 0.01; ***p < 0.001

Next, we tested the effect of MCPyV LT and sT on the transcriptional activity of HPV16 LCR and HPV18 in these three cell lines. Dose-dependent studies with expression plasmids for LT or sT or LT plus sT showed that LT as well as sT induced the activities of the HPV16 and HPV18 LCR in C33A cells (Fig. 2A). Transactivation of the HPV16 LCR and HPV18 LCR by MCPyV LT, but not sT was observed in HSC-3 cells (Fig. 2B) and HaCaT cells (Fig. 2C). In HaCaT cells, sT repressed rather that stimulated the transcriptional activities of the HPV16 LCR and HPV18 LCR. At higher concentrations (1200 ng) of empty, LT, or sT expression plasmids, reduction in the transcriptional activity of HPV16 LCR and HPV18 LCR was monitored probably because of promoter interference of the CMV promoter with the HPV16 and HPV18 LCRs (results not shown).

### Transactivation of the MCPyV early and late promoter activity by HPV16 E6 and E7

**Fig. 3:**
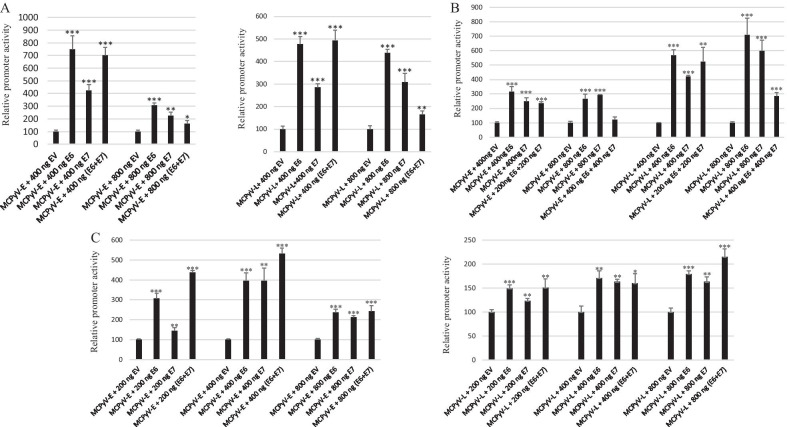
HPV16 E6 and E7 stimulates the transcriptional activity of the MCPyV early and late promoter. **A** C33A cells were transfected with 400 ng luciferase reporter plasmid and increasing amounts (400 ng or 800 ng) of expression plasmids for HPV16 E6 and E7. **B** HSC-3 cells were transfected with 400 ng luciferase reporter plasmid and increasing amounts of expression plasmids for HPV16 E6 and E7. **C** HaCaT cells were transfected with 400 ng luciferase reporter plasmid and increasing amounts of expression plasmids for HPV16 E6 and E7. Luciferase activity was corrected for the protein concentration in each sample. Each bar represents the average of three independent parallels ± SD. The transcriptional activity of the early (respectively late) promoter in the presence of empty vector pcDNA3.1 (EV) was arbitrary set as 100%. Similar results were obtained in an independent experiment. *p < 0.05; **p < 0.01; ***p < 0.001

As co-infection between HR-HPV and MCPyV has been reported, we investigated whether the HPV oncoproteins E6 and E7 had an effect on the MCPyV early and late promoter activity in C33A, HaCaT and HSC-3 cells. E6 and E7 increased the MCPyV early and late promoter activity in all three cell lines. No additive effect was observed when E6 and E7 were co-expressed (Fig. 3).

### Functional domains in LT and sT required for transactivation

**Fig. 4:**
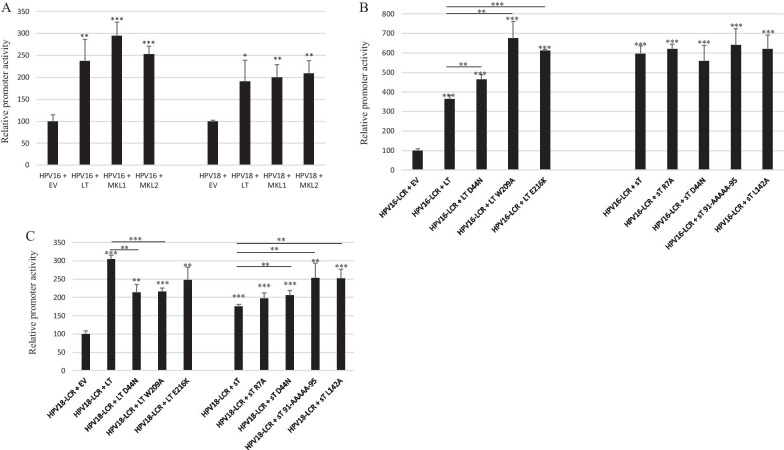
Effect of LT and sT mutants on HPV16 and HPV18 LCR activity. **A** MCPyV MKL-1 and MKL-2 truncated LT stimulate the transcriptional activity of the HPV16 LCR and HPV18 LCR in C33A cells. **B** Effect of MCPyV LT and sT mutants on the transcriptional activity of the HPV16 LCR. **C** Effect of MCPyV LT and sT mutants on the transcriptional activity of the HPV18 LCR. Cells were transfected with 400 ng luciferase reporter plasmid and 400 ng of expression plasmids for MCPyV full-length LT, MKL-1 LT, MKL-2 LT, LT mutants, or sT mutants. Luciferase activity was corrected for the protein concentration in the lysate. Each bar represents the average of three independent parallels ± SD. The transcriptional activity of the early (respectively late) promoter in the presence of empty vector pcDNA3.1 (EV) was arbitrary set as 100%. Similar results were obtained in an independent experiment. *p < 0.05; **p < 0.01; ***p < 0.001

To determine which function domain(s) of LT and sT were required for transactivation of the transcriptional activity of HPV16 and HPV18 LCR, we generated different mutants of these two MCPyV oncoproteins. Because expression of a truncated LT is a hallmark of MCPyV-positive Merkel cell carcinomas [41], we examined the effect of two such truncated LT on the transcriptional activity of HPV16 LCR and HPV18 LCR in C33A cells. The truncated MKL-1 and MKL-2 LT were used for this purpose. While full-length MCPyV LT is 817 amino acids long, MKL-1 and MKL-2 consist of 330 and 275 amino acids, respectively, removing the p53-binding motif and the DNA-binding domain. MKL-2 LT also lacks the nuclear localization signal (NLS) [42]. Induction of the transcriptional activity of HPV16 LCR and HPV18 LCR by MKL-1 and MKL-2 was comparable to full-length LT (Fig. 4A). This indicates that neither DNA binding nor nuclear localization of LT is required to stimulate the LCR transcriptional activity of HPV16 and HPV18. We then generated LT mutants in its DnaJ domain (D44N), the hVam6p binding domain (W209A), and the pRb binding pocket (E216K) [41, 43, 44]. All of these mutants were able to stimulate the HPV16 and HPV18 LCR transcriptional activity significantly more potent than wild-type LT (Fig. 4B, C). Western blot analysis showed that the mutants were expressed at similar levels compared to wild-type LT (Additional file 1: Fig. S1). These results indicate that LT-mediated induction of the HPC16 LCR and HPV18 LCR activity is independent of the ability of LT to interact with HSC70, pRb, and hVam6p.

Studies with sT mutants in either the DnaJ domain (D44N), the protein phosphatase 2A (PP2A) binding domains (R7A and L142A) and in the Fbxw7 binding domain (91-AAAAA-95) [43, 45, 46] demonstrated that these mutants enhanced HPV16 and HPV18 LCR transcriptional activity equally well or better than wild-type sT (Fig. 4B, C). These results suggest that sT-mediated induction of the HPC16 LCR and HPV18 LCR activity is independent of the interaction with HSC70, PP2A, and the ubiquitin ligase Fbxw7.

### Functional domains in E6 and E7 required for transactivation

**Fig. 5:**
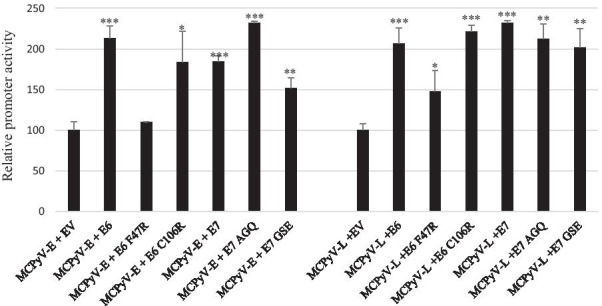
Effect of mutations in E6 and E7 on their ability to transactivate the early and late promoter of MCPyV. C33A cells were transfected with 400 ng luciferase reporter plasmid containing either the early or the late promoter of MCPyV. Co-transfection was done with 400 ng of empty expression vector (EV) or expression plasmids for wild-type LT, wild-type sT or their mutants. Luciferase activity was corrected for the protein concentration in the lysate. Each bar represents the average of three independent parallels ± SD. The transcriptional activity of the early (respectively late) promoter in the presence of empty vector pcDNA3.1 (EV) was arbitrary set as 100%. Similar results were obtained in an independent experiment. *p < 0.05; **p < 0.01; ***p < 0.001

Next, the functional domains of E6 and E7 required for induction of the MCPyV early and late promoter activity were characterized. We created a HPV16 E6 mutant with substitution of residue 47, which is phenylalanine into arginine and a mutant in which cysteine at position 106 was replaced by arginine (numbering according to protein accession number AYV61474). This E6 F47R mutant is defective for ubiquitination and subsequent degradation of p53 and the C106R mutation abolishes binding to p53 binding [47, 48]. For HPV16 E7 we constructed a mutant in the pRb pocket motif LYCYE, which was replaced by AYGYQ, and a mutant in which the CCKC sequence was changed into GSEC. The E7 AGQ mutant is unable to bind pRb [49]. The CCKC motif is assumed to be important for maintaining the correct configuration of E7 [50]. All the residues that were mutated in HPV16 E6 and HPV E7 are conserved in the corresponding proteins of HPV18 (F49 and C108 for E6 and LLCHE and CCKC for E7; protein accession numbers ATL15239 and ATL15240; Additional file 2: Fig. S2). All these mutations have been shown to reduce the transactivation property of HPV16 E6 and E7, respectively [12, 47–49]. Our results showed that only the mutation F47R reduced E6-induced activation of the MCPyV promoters (Fig. 5).

### Transactivation of HPV16 and HPV18 LCR deletion mutants by LT and sT

**Fig. 6:**
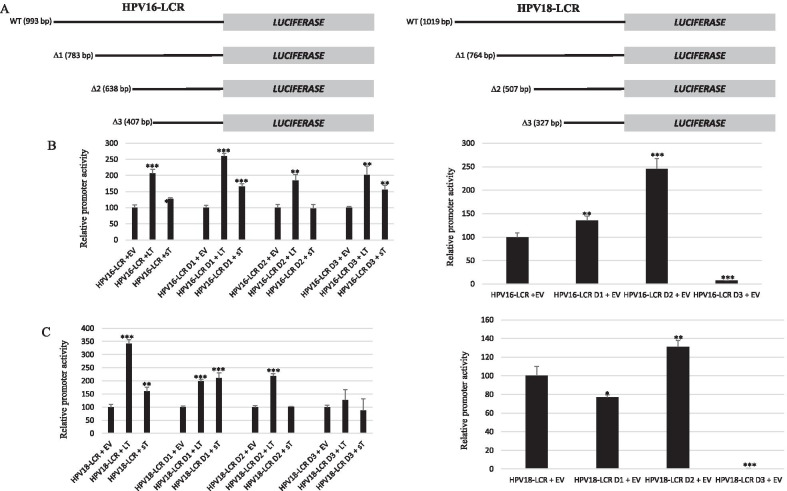
Effect of MCPyV LT and sT on the transcriptional activity of HPV16 LCR and HPV18 LCR mutants in C33A cells. **A** Schematic presentation of the truncated HPV16 and HPV18 LCR mutants. An additional SacI site was introduced by site-directed mutagenesis in the luciferase reporter plasmid containing the HPV16 LCR or the HPV18 LCR. The mutated plasmid was then cut with SacI and religated, resulting in truncation of the distal sequences of the LCR. The number of nucleotides in the LCR is given in parenthesis. **B** Cells were co-transfected with 400 ng luciferase reporter plasmid containing the HPV16 LCR or truncated versions and 400 ng of expression plasmids for LT or sT. The figure in the left panel shows transactivation by LT or sT. The transcriptional activity of the early (respectively late) promoter in the presence of empty vector pcDNA3.1 (EV) was arbitrary set as 100%. The figure in the right panel represents the activity of HPV16 LCR and its truncated versions. The activity of the full-length HPV16 LCR was arbitrary set as 100%. **C** As in (**B**) but with the luciferase reporter plasmid with HPV18 LCR or truncated versions. Luciferase activity was corrected for the protein concentration in the lysate. Each bar represent the average of three independent parallels ± SD. Similar results were obtained in an independent experiment. *p < 0.05; **p < 0.01; ***p < 0.001

To map the sequences required for LT- and sT-mediated activation of the HPV16 LCR and HPV18 LCR, deletion mutants were generated. Truncation of the 5′ region and part of the central region of the HPV16 LCR (Δ1 and Δ2, see Fig. 6) increased the basal transcriptional activity. Additional deletion of the central region strongly reduced basal HPV16 LCR activity. The transcriptional activity of all truncated LCRs was still induced by LT and sT. For the HPV18 LCR, deletion of the distal part of the promoter (Δ1 mutant) reduced basal promoter activity by approximately 20%, while additional deletion of part of the central domain of the LCR (Δ2) increased basal promoter activity by ~ 25%. The activity of these truncated LCR was still induced by LT and sT. The LCR fragment containing only the 3′ LCR sequences (Δ3) had < 10% promoter activity compared to full-length LCR and was not induced by LT or sT.

### Transactivation of the MCPyV early and late promoter deletion mutants by E6 and E7

**Fig. 7:**
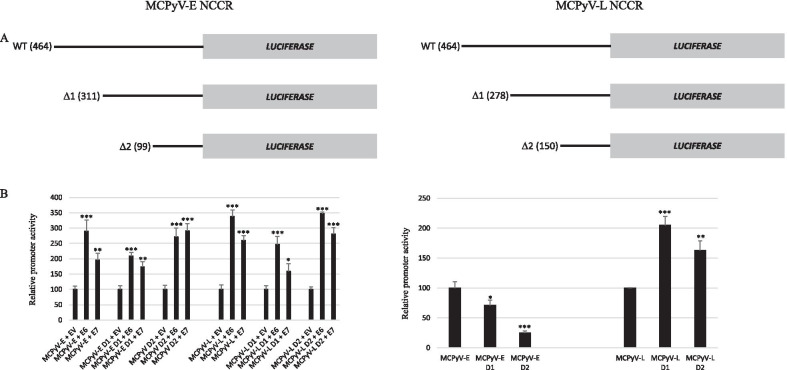
Effect of E6 and E7 on the transcriptional activity of MCPyV early and late promoter deletion mutants in C33A cells. **A** Schematic presentation of the full length and truncated MCPyV early and late promoter. The non-coding region (NCCR) consists of 464 bp was cloned in late to early direction upstream of the *luciferase* gene (= early MCPyV promoter) or in the early to late direction upstream of the *luciferase* gene (= late MCPyV promoter). The number of nucleotides in the promoter is given in parenthesis. **B** Cells were co-transfected with 400 ng luciferase reporter plasmid containing the MCPyV early (respectively late) promoter or truncated versions and 400 ng of expression plasmids for E6 or E7. The figure in the left panel shows transactivation by E6 or E7. The transcriptional activity of the early (respectively late) promoter in the presence of empty vector pcDNA3.1 (EV) was arbitrary set as 100%. The figure in the right panel represents the activity of early promoter (respectively late promoter) and its truncated versions. The activity of the full-length MCPyV early promoter (respectively late promoter) was arbitrary set as 100%. Luciferase activity was corrected for the protein concentration in the lysate. Each bar represent the average of three independent parallels ± SD. Similar results were obtained in an independent experiment. *p < 0.05; **p < 0.01; ***p < 0.001

To determine which sequences were necessary for the induction of MCPyV early and late promoter activity by E6 and E7, truncated mutants were produced (Fig. 7A). None of the deletions affected the abilities of E6 and E7 to stimulate the early and the late promoter activity (Fig. 7B). These results suggest that E6 and E7 may mediate increased MCPyV promoter activity through the basic transcription machinery rather than specific transcription factors. Expanding truncation gradually reduced the basal activity of the MCPyV early promoter, but increased basal late promoter activity (Fig. 7B). The NCCR may contain sequences on the early region site that have a negative effect on the late promoter.

### Effect of LT and sT on E6 and E7 protein expression

**Fig. 8:**
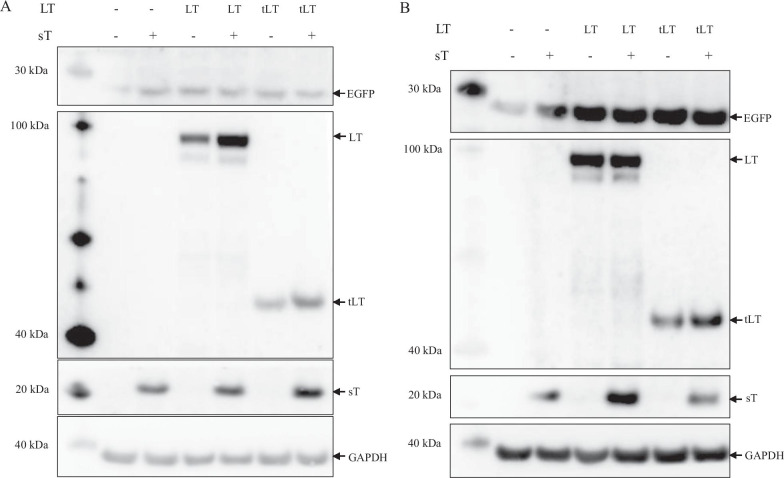
MCPyV LT and sT stimulate HPV16 LCR and HPV 18 LCR-driven protein expression. HaCaT cells stably transfected with EGFP expression plasmid containing the HPV16 LCR or the HPV 18 LCR were transfected with expression plasmid for MCPyV LT, sT, truncated LT variant MKL-2 (tLT) or a combination of LT (respectively tLT) plus sT. Lysates were prepared 24 h after transfection and expression of EGFP, LT, sT, tLT, and GAPDH was monitored using antibodies. The lane on the left represents the protein marker (in kDa). **A** HaCaT cells stably expressing EGFP under control of HPV16 LCR. **B** HaCaT cells stably expressing EGFP driven by the HPV18 LCR. The top panel of the figure shows EGFP protein expression. The middle panels confirm the expression of LT, truncated LT and sT, respectively. The loading control GAPDH is shown in the bottom panel

Stimulation of the HPV16 LCR and HPV18 transcriptional activity by LT and sT urged us to investigate whether LT and sT could increase expression levels of the E6 and E7 oncoproteins of these two HR-HPV. For this purpose, we transfected SiHa cells (HPV16 positive) and Hela cells (HPV18 positive) with empty expression vector or expression plasmids for LT, sT, truncated LT variant MKL-2 or a combination of LT + sT or MKL-2 + sT and monitored E6 and E7 expression levels using western blot 24 and 48 h after transfection. However, inconsistent and irreproducible results were obtained with different antibodies. Moreover, E6 and E7 expression levels were very low in SiHa cells (results not shown). This is in agreement with the relatively low HPV16 LCR activity in C33A cells (Fig. 1). Because of the technical problems and the fact that the transcriptional activity of both HPV16 LCR and HPV18 LCR was high in HaCaT cells (Fig. 1) and keratinocytes are often used for HPV studies, we generated HaCaT cell lines that stably expressed EGFP either under control of the HPV16 LCR or under control of the HPV18 LCR. These cell lines were transfected with expression plasmids for LT, sT, MKL-2, LT plus sT, or MKL-2 plus sT and the expression levels of HPV16 LCR- and HPV18 LCR-directed EGFP were monitored. A strong increase in HPV16 and HPV18 LCR driven EGFP expression was observed in the presence of LT, sT. MKL-2, and co-presence of LT + sT or MKL-2 + sT (Fig. 8A, B). These results confirm that LT and sT increase protein levels of genes under control of the HPV16 LCR or the HPV18 LCR.

### Effect of E6 and E7 on LT protein levels

**Fig. 9:**
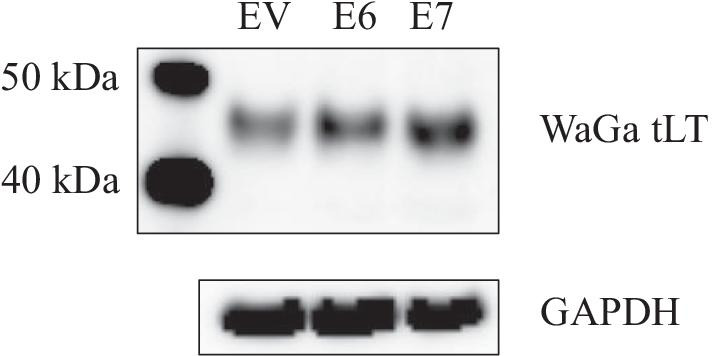
HPV16 E6 and E7 stimulate expression of MCPyV LT. MCPyV-positive WaGa cells were transfected with empty vector (EV) or plasmids coding for HPV16 E6 or HPV16 E7. Cell lysates were prepared 24 h after transfection and LT expression levels were analyzed by western blotting. GAPDH was used as a loading control. The molecular mass marker (in kDa) is shown in the lane on the left

Because E6 and E7 stimulated the MCPyV early promoter in a luciferase reporter assay, we wanted to examine whether E6 and E7 could also induce the expression of the MCPyV early protein LT. For this purpose, the MCPyV-positive MCC line WaGa was transfected with expression plasmids for HPV16 E6 or E7. WaGa cells expressed a truncated version of LT of ~ 40–45 kDa, which is typical for virus-positive MCC cells [51]. Transfecting these cells with expression plasmid of E6 or E7 resulted in increased protein levels of LT, supporting our findings that E6 and E7 stimulate MCPyV early promoter activity (Fig. 9). As no viral late proteins are expressed in virus-positive MCC cells, the effect of E6 and E7 on the protein expression driven by the MCPyV late promoter could not be tested.

## Discussion

A causal role of HR-HPV, especially HPV16 and HPV18, in anogenital and oropharyngeal cancers is well-established [2, 3, 5–8]. MCPyV is the etiological factor of 80% of MCC [15], but MCPyV has also been detected in oropharyngeal and anogenital tumors, but their role in these cancers is not known (reviewed in [52]). Because double infection with HR-HPV and MCPyV has been reported in anogenital and oropharyngeal cancers [24, 32], we examined whether an interaction between HR-HPV and MCPyV exists.

First, we compared the promoter activities of HPV16, HPV18 and MCPyV in three different cell lines. We found that the transcriptional activity of the HPV18 LCR was 6–10 times stronger than that of the HVP16 LCR in C33A and HaCaT cells, but comparable in HSC-3 cells (Fig. 1). Chen et al*.* also reported that the basal transcriptional activity of the HPV18 LCR was about three fold higher than that of HPV16 LCR in HeLa cells and in breast cancer T47D cells [53]. Ottinger et al*.* showed that the HPV18 LCR activity was 5–20-fold stronger than the HPV16 LCR activity in the cervical carcinoma cell lines C33A, HeLa and SiHa and in normal oral keratinocytes, but lower in African Green Monkey kidney epithelial cells CV-1 and comparable in hTERT immortalized human BJ fibroblasts [54]. The MCPyV early and late promoter activities were comparable in C33A and HaCaT cells, whereas the early promoter was approximately 40% stronger in HSC-3 cells. The early as well as the late MCPyV promoter had significantly weaker transcriptional activity than the HPV16 LCR and the HPV18 LCR in HaCaT and HSC-3 cells, but both were stronger compared to HPV16 LCR in C33A. To our best knowledge, this is the first study comparing the basal activities of the HPV16/18 LCRs and MCPyV promoter. Transient expression of MCPyV sT, full-length LT, or sT plus LT was shown to stimulate the transcriptional activity of the HPV16 and HPV18 LCR in C33A and HSC-3 cells, whereas LT induced HPV16 LCR and HPV18 LCR activity in HaCaT cells, but not sT, which significantly reduced the activity of these LCRs in HaCaT cells (Fig. 2). These results suggest a cell-specific effect of LT and sT on the LCR activity of HPV16 and HPV18. Previous studies with the HPV16 and HPV18 promoter in other cell lines showed a variable stimulating effect of LT and sT of the polyomavirus SV40. SV40 LT increased HPV18 promoter activity 13-fold in HeLa cells, ~ threefold in SW13 (human adrenocortical carcinoma) and in 3T6 (mouse fibroblasts) cells, but had no effect in monkey kidney CV-1 cells [55]. In human keratinocytes, a ninefold increase in HPV18 promoter strength was observed in the simultaneous presence of LT and sT [56]. In human embryonal fibroblasts, the HPV16 promoter activity was stimulated 20- to 30-fold by SV40 sT, while the effect of LT was 5- to sixfold weaker than sT [57]. We examined the mechanism by which MCPyV LT and sT stimulate the activity of the LCR of HPV16 and HPV18. LT can activate heterogeneous promoters by binding to adjacently repeated G^A^/_G_GGC motifs [58]. Yet, a sequence analysis of the HPV16 and HPV18 promoters did not reveal repeated LT binding motifs. Moreover, the C-terminal truncated LT variants MKL1 and MKL2, which lack the DNA binding domain, still activated the HPV16 and HPV18 promoters (Fig. 4A). Therefore, the nuclear presence of LT seems not necessary because the MKL2 LT variant lacks a functional NLS [42]. Mutations in LT that abrogate the binding to HSC70, pRb and to hVam6p did not impair LT-mediated *trans*-activation of the HPV 16 LCR and HPV18 LCR. Likewise, sT mutants defective in interacting with HSC70, PP2A, and ubiquitin ligase Fbxw7 did not abolish sT-induced stimulation of HPV16 LCR and HPV18 LCR transcriptional activity (Fig. 4B, C). The exact mechanism by which the MCPyV oncoproteins stimulate HVP16 and HPV18 LCR activity remains unknown. LT and sT can affect different signaling pathways [19, 59], which may regulate the activity of transcription factors that control the transcriptional activity of these LCRs. The exact sequences required for LT- and sT-mediated activation of the HPV16 LCR and HPV18 LCR could not be determined by truncation versions of these LCRs because the transcriptional activity of truncated LCRs was still induced by LT and sT. Further studies are required to map the exact LCR sequences that mediate LT- and sT-induced activation. The LCR of the HPV16 and HPV18 contain binding motifs for several transcription factors, including AP1, NF1, YY1, SP1, OCT-1, TEF-1, C/EBPβ, STAT3, glucocorticoid/progesterone receptor, FOXA1, MYC, and some distinctive ones such as SOX2 and FOXA-2 for HPV16 LCR and GATA3 and TFAB2B for HPV18 LCR [60–62], but it remains to be determined whether these are implicated in LT- or sT-triggered activation of the HPV16/18 LCR. MCPyV LT and sT can interact with several proteins involved in signaling and transcription (reviewed in [19]), but the biological roles of these interactions are unknown. Stimulation of the MCPyV early and late promoters by E6 and E7 was only affected by the E6 F47R mutant. This E6 mutant, which counteracts p53 degradation mediated by wild-type E6 [48], failed or had reduced ability to stimulate the MCPyV early and late promoter. However, the E6 C106R mutant, which cannot bind p53, increased MCPyV early and late promoter activity comparable to wild-type E6. More than 50 different cellular proteins interact with E6 [11, 13], but whether the F47R mutation disrupts such interaction(s) thereby affecting E6’s ability to trans-activate the MCPyV promoters remains to be established.

## Conclusions

Our results show that the oncoproteins LT and sT from MCPyV can stimulate the transcriptional activity of the HPV16 LCR and the HPV18 LCR and induce expression of proteins from these LCR. On the other hand, the E6 and E7 oncoproteins of HR-HPV can trans-activate the MCPyV early and late promoter and augment expression of the MCPyV LT oncoprotein. The exact mechanism for this reciprocal trans-activation remains elusive. Co-infection with HR-HPV and MCPyV may enhance the oncogenic potentials of these viruses through reciprocal potentiating the expression levels of their oncoproteins.

## Supplementary Information

**Additional file 1: Fig. S1**. Expression of the MCPyV LT and sT mutants. Cells were co-transfected with the empty expression vector pcDNA3.1 (EV) or and expression plasmid for wild type or mutant LT or sT and with a plasmid encoding GFP. Protein expression was monitored using antibodies against MCPyV LT, FLAG (FLAG-tagged sT) and EGFP. EGFP was used to validate for transfection efficiency and GAPDH was used as a loading control. The protein marker (in kDa) is shown in the last lane.

**Additional file 2: Fig. S2**. Amino acid sequence alignment of the E6 and E7 proteins of HPV16 and HPV18. The amino acids that were mutated in HPV16 E6 and E7 are highlighted and are conserved in HPV18 E6 and E7.

## Data Availability

All data generated or analyzed during this study are included in this published article.

## Abbreviations

BKPyV: BK polyomavirus
CMV: Cytomegalovirus
HPV: Human papillomavirus
HR-HPV: High-risk human papillomavirus
LCR: Long control region
LT: Large tumor antigen
MCC: Merkel cell carcinoma
MPyV: Merkel cell polyomavirus
NLS: Nuclear localization signal
PP2A: Protein phosphatase 2A
sT: Small tumor antigen
