# Supplementary material for: Reciprocal transactivation of Merkel cell polyomavirus and high-risk human papillomavirus promoter activities and increased expression of their oncoproteins
Source: Virol J. 2021 Jul 3;18:139. doi: 10.1186/s12985-021-01613-0 (PMC8254899; doi:10.1186/s12985-021-01613-0)

Supplementary Figure S1. Expression of the MCPyV LT and sT mutants. Cells were co-transfected with the empty expression vector pcDNA3.1 (EV) or and expression plasmid for wild type or mutant LT or sT and with a plasmid encoding GFP. Protein expression was monitored using antibodies against MCPyV LT, FLAG (FLAG-tagged sT) and EGFP. EGFP was used to validate for transfection efficiency and GAPDH was used as a loading control. The protein marker (in kDa) is shown in the last lane.

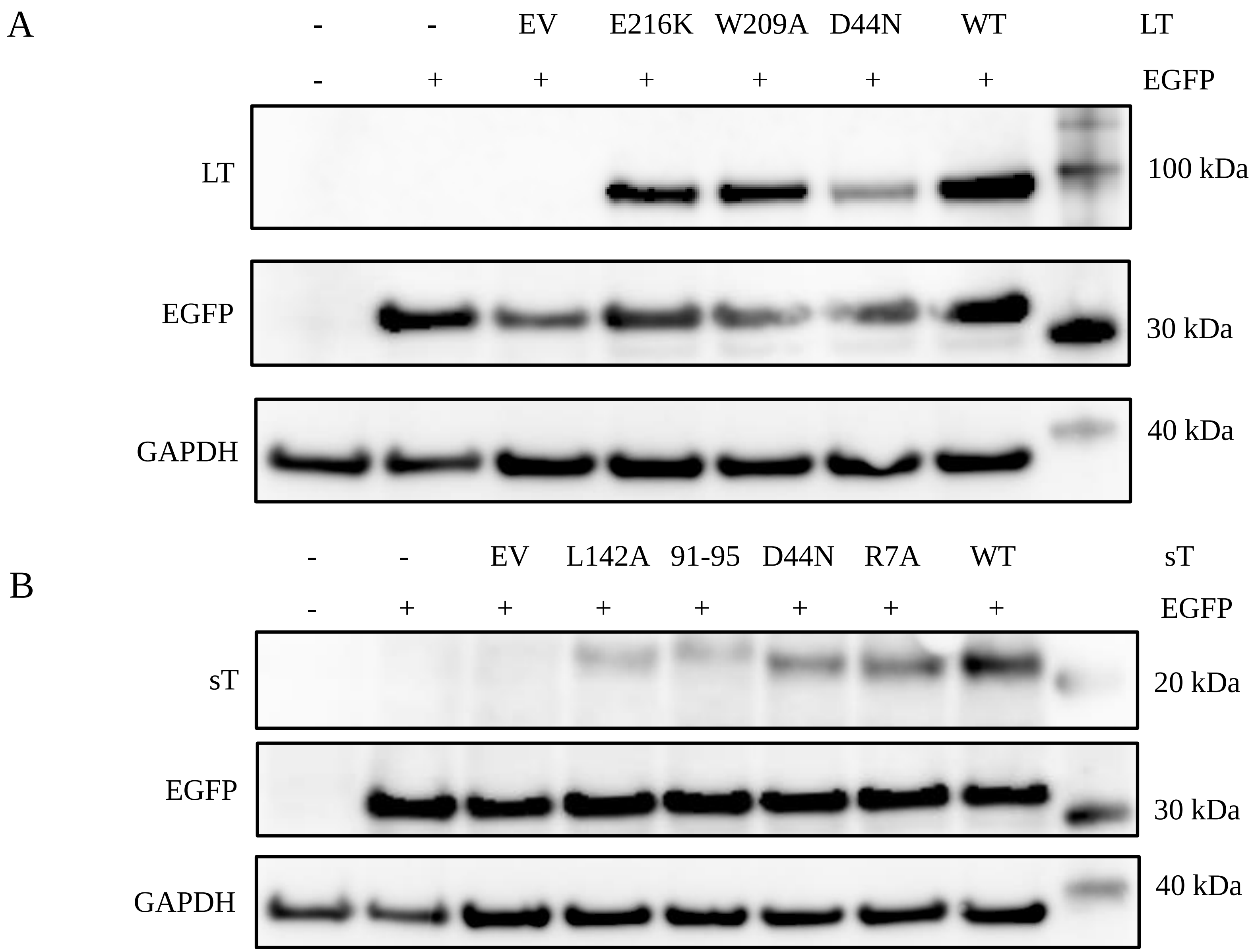

Supplement: Supplementary file 1 — Additional file 1: Fig. S1. Expression of the MCPyV LT and sT mutants. Cells were co-transfected with the empty expression vector pcDNA3.1 (EV) or and expression plasmid for wild type or mutant LT or sT and with a plasmid encoding GFP. Protein expression was monitored using antibodies against MCPyV LT, FLAG (FLAG-tagged sT) and EGFP. EGFP was used to validate for transfection efficiency and GAPDH was used as a loading control. The protein marker (in kDa) is shown in the last lane. [file 12985_2021_1613_MOESM1_ESM.pdf]
