# Supplementary material for: Reciprocal transactivation of Merkel cell polyomavirus and high-risk human papillomavirus promoter activities and increased expression of their oncoproteins
Source: Virol J. 2021 Jul 3;18:139. doi: 10.1186/s12985-021-01613-0 (PMC8254899; doi:10.1186/s12985-021-01613-0)
Supplement: Supplementary file 2 — Additional file 2: Fig. S2. Amino acid sequence alignment of the E6 and E7 proteins of HPV16 and HPV18. The amino acids that were mutated in HPV16 E6 and E7 are highlighted and are conserved in HPV18 E6 and E7. [file 12985_2021_1613_MOESM2_ESM.pdf]

**Supplementary Figure S2.** Amino acid sequence alignment of the E6 and E7 proteins of HPV16 and HPV18. The amino acids that were mutated in HPV16 E6 and E7 are highlighted and are conserved in HPV18 E6 and E7.

**HPV16 E6 (AYV61474); 158 aa**

MHQKRTAMFQDPQERPGKLPQLCTELQTTIHDIILECVYCKQQLLRREVYDFA**F**RDLCIV
YRDGNPYAVCDKCLKFYSKISEYRHYCYSVYGTTLEQQYNKPLCDLLIRCIN**C**QKPLCPE
EKQRHLDKKQRFHNIRGRWTGRCMSCCRSSRTRRETQL

**HPV18 E6 (ATL15239); 158 aa**

MARFEDPTRRPYKLPDLCTELNTSLQDIEITCVYCKTVLELTEVFEFA**F**KDLFVVYRDSI
PHAACHKCIDFYSRIRELRHYSDSVYGDTLEKLTNTGLYNLLIRCLR**C**QKPLNPAEKLRH
LNEKRRFHNIAGHYRGQCHSCCNRARQERLQRRRETQV

```
HPV16      MHQKRTAMFQDPQERPGKLPQLCTELQTTIHDIILECVYCKQQLLRREVYDFAFRDLCIV      60
HPV18      -----MARFEDPTRRPYKLPDLCTELNTSLQDIEITCVYCKTVLELTEVFEFAFKDLFVV      55
                 * *:** .** ***:*****:*:::** : *****  *   **::***:** :*
HPV16      YRDGNPYAVCDKCLKFYSKISEYRHYCYSVYGTTLEQQYNKPLCDLLIRCINCQKPLCPE      120
HPV18      YRDSIPHAACHKCIDFYSRIRELRHYSDSVYGDTLEKLTNTGLYNLLIRCLRCQKPLNPA      115
           ***. *:*.*.**:.***:* * ***. **** ***:  *. * :*****:.***** *
HPV16      EKQRHLDKKQRFHNIRGRWTGRCMSCCRSSRT-----RRETQL      158
HPV18      EKLRHLNEKRRFHNIAGHYRGQCHSCCNRARQERLQRRRETQV      158
           ** ***::*:***** *:: *:* ***. :* .*  :*****
```

**HPV16 E7 (AYV61475), 98 aa**

MHGDTPTLHEYMLDLQPETTD**L**Y**C**Y**E**QLNDSSEEEDEIDGPAGQAEPDRAHYNIVTF**CCK**
CDSTLRLCVQSTHVDIRTLEDLLMGTLGIVCPICSQKP

**HPV18 E7 (ATL15240); 105 aa**

MHGPKATVQDIVLHLEPQNEIPVD**L**L**C**H**E**QLSDSEEENDEIDGVNHQHLPARRAEPQRHT
LLCM**CCK**CEARIELVVESSADDLRAFQQLFLNTLSFVCPWCASQQ

```
HPV16      MHGDTPTLHEYMLDLQPE---TTDLYCYEQLNDSSEEEDEIDGPAGQAEPDRA----HYN      53
HPV18      MHGPKATVQDIVLHLEPQNEIPVDLLCHEQLSDSEEENDEIDGVNHQHLPARRAEPQRHT      60
           ***  . *::: :*.*:*:    .** *:***.**.**:*****    *  * *     ::.
HPV16      IVTFCCKCDSTLRLCVQSTHVDIRTLEDLLMGTLGIVCPICSQKP    98
HPV18      LLCMCCKCEARIELVVESSADDLRAFQQLFLNTLSFVCPWCASQQ    105
           :: :****:: :.* *:*:  *:*::::*::.**.:*** *:.:
```
